# Supplementary material for: Electronic Polarizability Tunes the Function of the Human Bestrophin 1 Cl– Channel
Source: J Chem Theory Comput. 2025 Jan 3;21(2):933–42. doi: 10.1021/acs.jctc.4c01039 (PMC11780730; doi:10.1021/acs.jctc.4c01039)
Supplement: Supplementary file 1 — ct4c01039_si_001.pdf [file ct4c01039_si_001.pdf]

Supplementary Information

Electronic Polarizability Tunes the Function of the Human Bestrophin 1 Cl^- Channel

Linda X. Phan[1,2], Aaron P. Owji[3,4,5], Tingting Yang[3], Jason Crain[2,6], Mark S.P. Sansom[2], and Stephen J. Tucker[1,7]*

[1] Clarendon Laboratory, Department of Physics, University of Oxford, Oxford, OX1 3PU, UK

[2] Department of Biochemistry, University of Oxford, Oxford, OX1 3QU, UK

[3] Department of Ophthalmology, Columbia University, New York, NY 10032, USA

[4] Department of Pharmacology, Columbia University, New York, NY 10032, USA

[5] Simons Electron Microscopy Center, New York Structural Biology Center, New York, NY 10027, USA

[6] IBM Research Europe, Hartree Centre, Daresbury, WA4 4AD, UK

[7] Kavli Institute for Nanoscience Discovery, University of Oxford, OX1 3QU, UK

* To whom correspondence should be addressed:

stephen.tucker@physics.ox.ac.uk

Supplementary Methodological Details

Full protein embedded simulations

The full protein systems were prepared using a multiscale procedure. The protein is first coarse-grained (CG) and then embedded into a POPC bilayer and solvating with water and ~ 0.5 M NaCl using Martini version 2.2 (1) and GROMACS 2021 package (2). This system is subject to 100 ns of equilibration in CG before being converted to atomistic representation with the CG2AT protocol (3). An equilibration period of 20 ns followed by a production run of 100 ns using the c36m forcefield (4) and mTIP3P water model was performed. The temperature was maintained at 310 K with coupling constant 1.0 ps by the Nosé-Hoover thermostat (5). Pressure was maintained at 1 bar with coupling constant 5.0 ps by the Parrinello-Rahman barostat (6). Short-range electrostatics were treated with the Verlet cutoff scheme at 1.2 nm cutoff and long-range electrostatics were treated with PME (7). C-alpha atoms were placed under harmonic restraints with a force constant of 1000 kJ/mol/nm^2 to prevent the structure from deviating too much from the experimental structure.

Figure S1

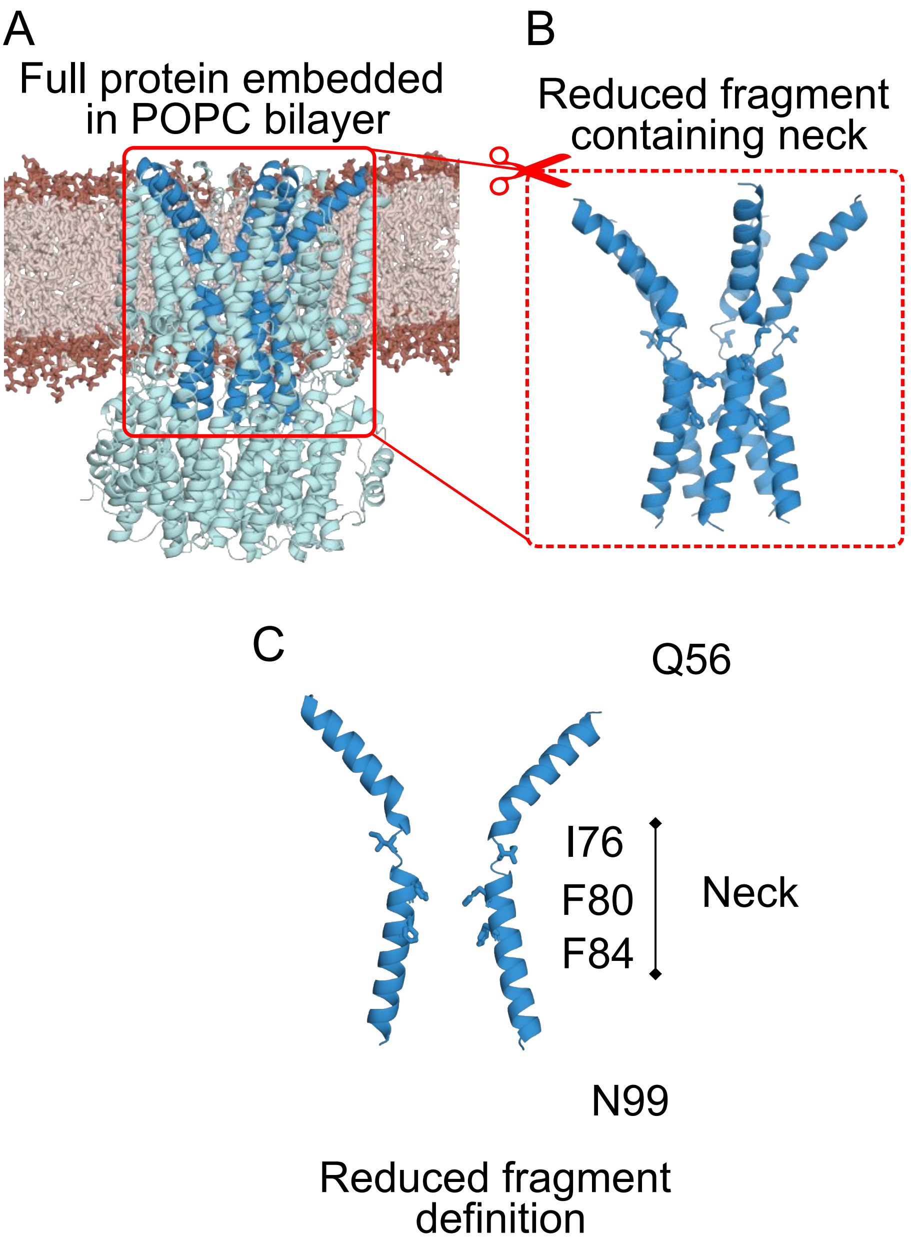

***Figure S1**:* **A** Full protein (cyan) embedded in POPC bilayer (brown). **B** Protein fragment system used in simulations due to the high computational demands of the AMOEBA forcefield. The full protein embedded system in solution contains ~200500 atoms. **C** The protein was truncated at Q56 and N99 such that the remaining fragment system contains the conserved hydrophobic neck region of interest (I76, F80, F84). The reduced protein fragment in solution contains ~57000 atoms. Water and ions are omitted for clarity.

Figure S2

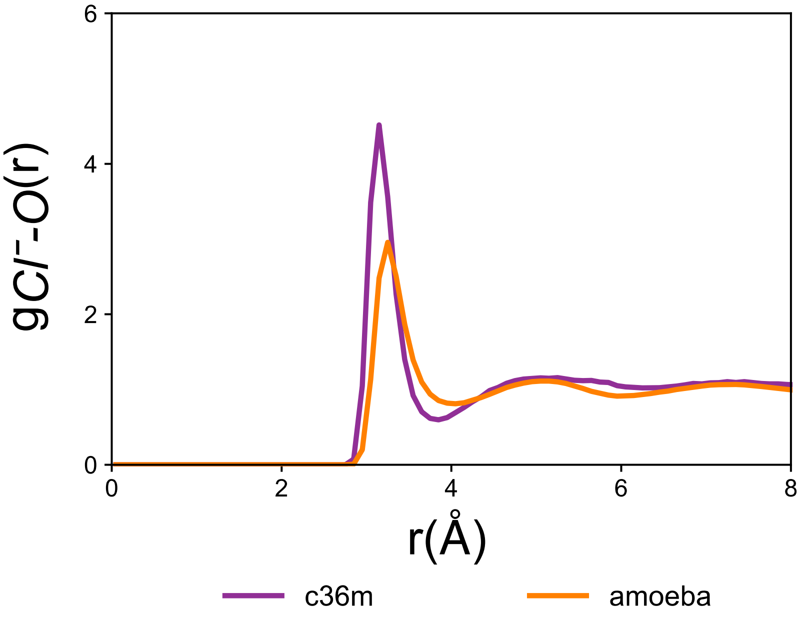

Figure S2: Radial distribution functions (RDFs), $g_{Cl^{-}-O}(r)$, of water oxygen atoms around a Cl^{-} ion in bulk solution with the c36m and AMOEBA forcefields.

Figure S3

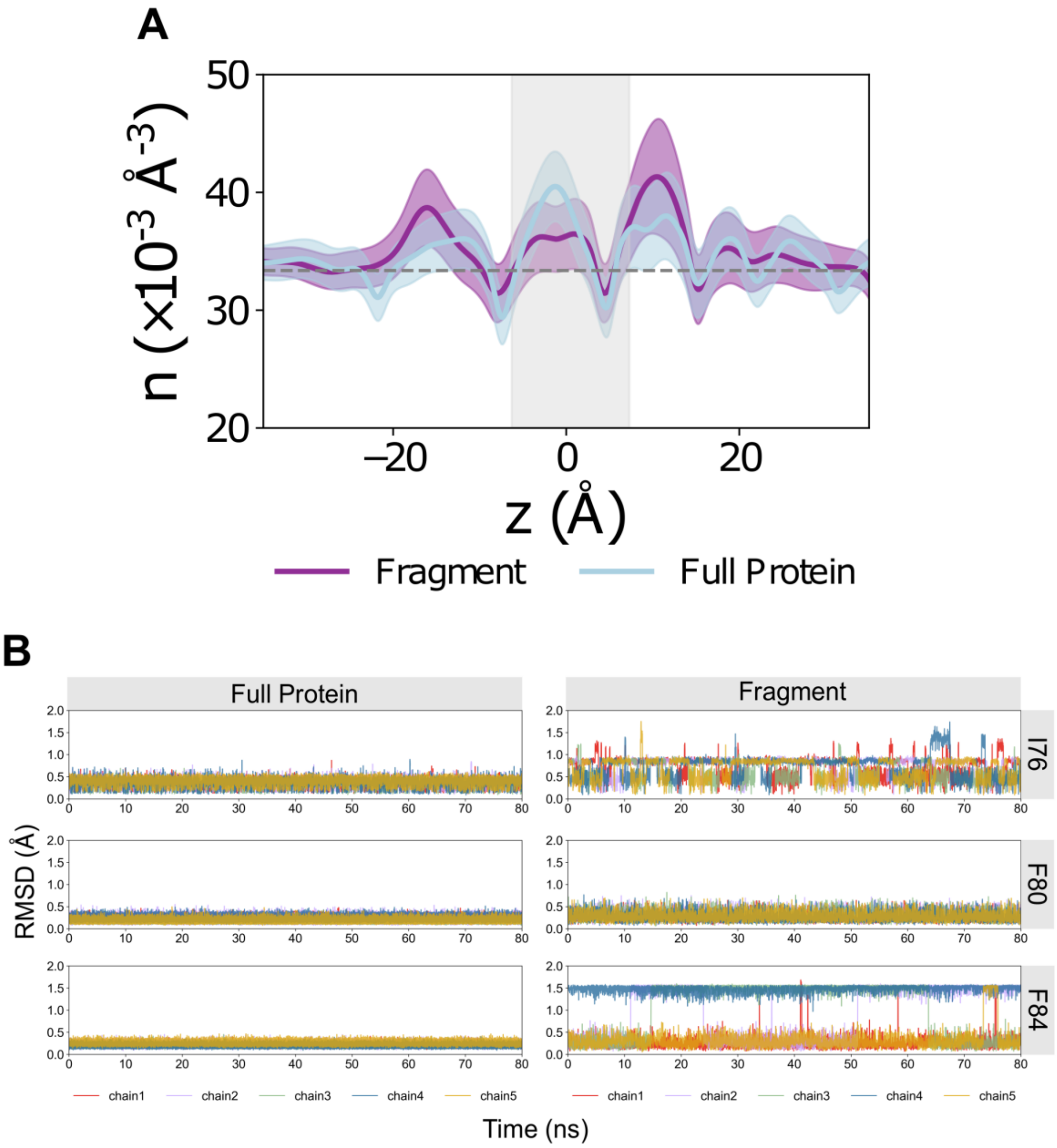

Figure S3: Protein fragment system validation. **A** Time-averaged water density profiles within the pore for the full protein embedded system (light blue) compared with the protein fragment in solution (purple) using c36m. The shaded region represents the neck region, and the dashed grey line corresponds to the density of bulk water (33.37 nm^{-3}). Confidence bands represent the standard deviation over the simulation. **B** RMSDs of pore-lining side chains of the neck residuesI76, F80 and F84 for simulations of the full protein system and the protein fragment. The full protein is very stable however the fragment sidechains are subject to fluctuations however all within <2 Å which are considered to be relatively stable.

Figure S4

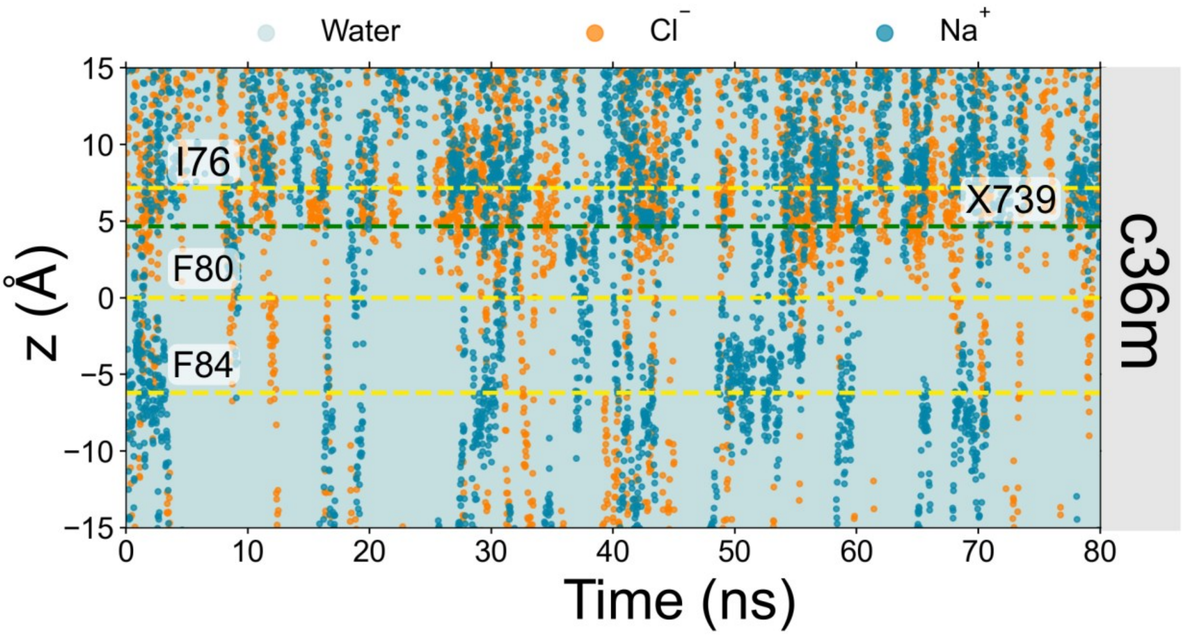

Figure S4: Trajectories of water (cyan), Na^+ (dark blue) and Cl^- (orange) ions in z-coordinates as a function of time within the neck region of the fragment protein in a c36m simulation. The green dashed line represents the location of X739 from the experimental structure and the plot indicates that simulations with the c36m forcefield show no clear accumulation of Cl^- in the region between I76 and F80, indicating there is no clear association with the binding site.

Figure S5

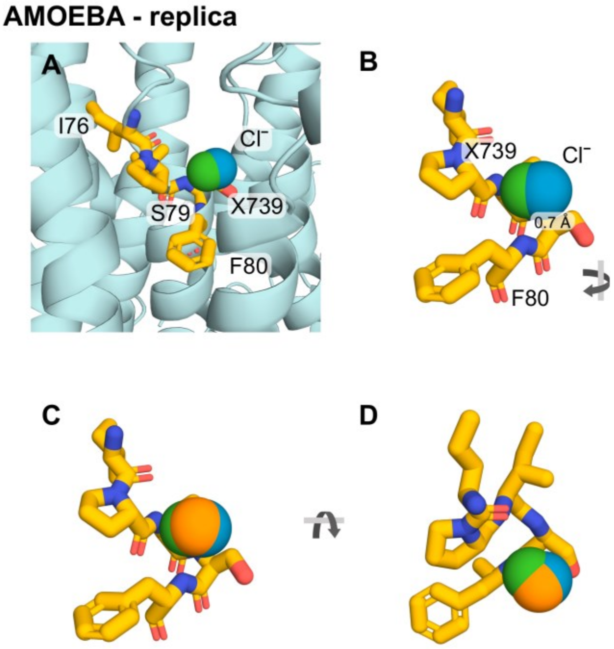

E

Forcefield	Site Residence Time (ns)	Duration (ns)	Occupancy (%)	R-squared	Distance to N of F80 (Å)	Distance to X739 in PDB (Å)
AMOEBA run1 (Figure 2)	0.22	0.31	47	0.9945	3.6	0.7
AMOEBA run2	0.25	0.32	68	0.9921	3.4	0.8
c36m (Figure 2)	0.06	-	15	-	6.0	4.5

Figure S5: AMOEBA simulation replica. **A** The top detected binding pose identified with PyLipID shows significant overlap comparable to the independent replicate shown in Figure 2 of the main manuscript. **B** The overlap between X739 (green sphere) and the detected Cl^- (blue sphere) is 0.7 Å. **C** illustrates the overlap between X739, Cl^- in this replica and Cl^- (orange sphere) from Figure 2 (first replica). Similarly, **D** shows a rotated view. **E** provides tabulated data associated with the observed binding sites in the AMOEBA simulations, AMOEBA replicate and c36m simulation. The c36m simulations exhibit very weak and transient interactions such that the binding durations have been too short to accurately measure. The Cl^- binding site was not detected in c36m simulation replicas (hence stats not shown here) supporting the interaction is very weak and transient and may not be considered as binding.

Figure S6

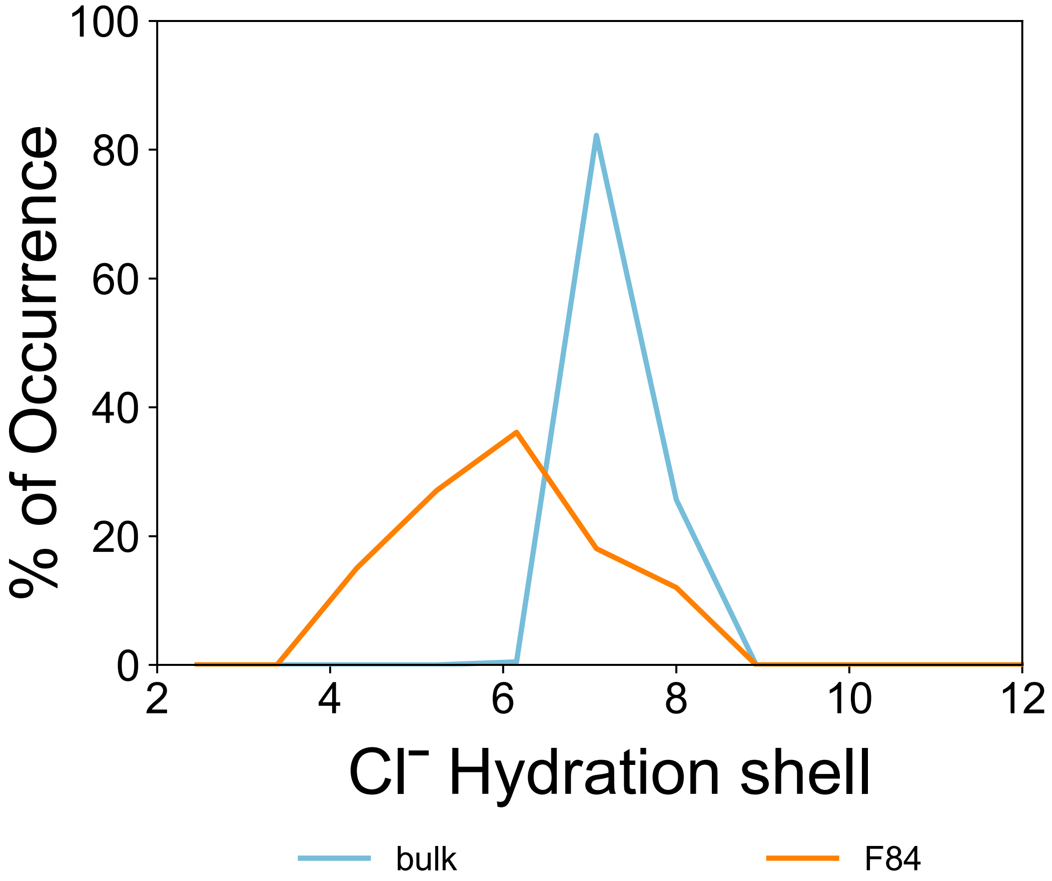

Figure S6: First hydration shell number of chloride ions in the partially open state structure (PDB ID 8D1K) in bulk (blue) compared with at the z-position corresponding to F84 (orange) within the pore in the AMOEBA simulation. Chloride loses 1-2 water molecules in its first hydration shell at F84 relative to bulk. This is comparable to the dehydration that occurs at F80 in the fully open state (PDB ID 8D1O).

Figure S7

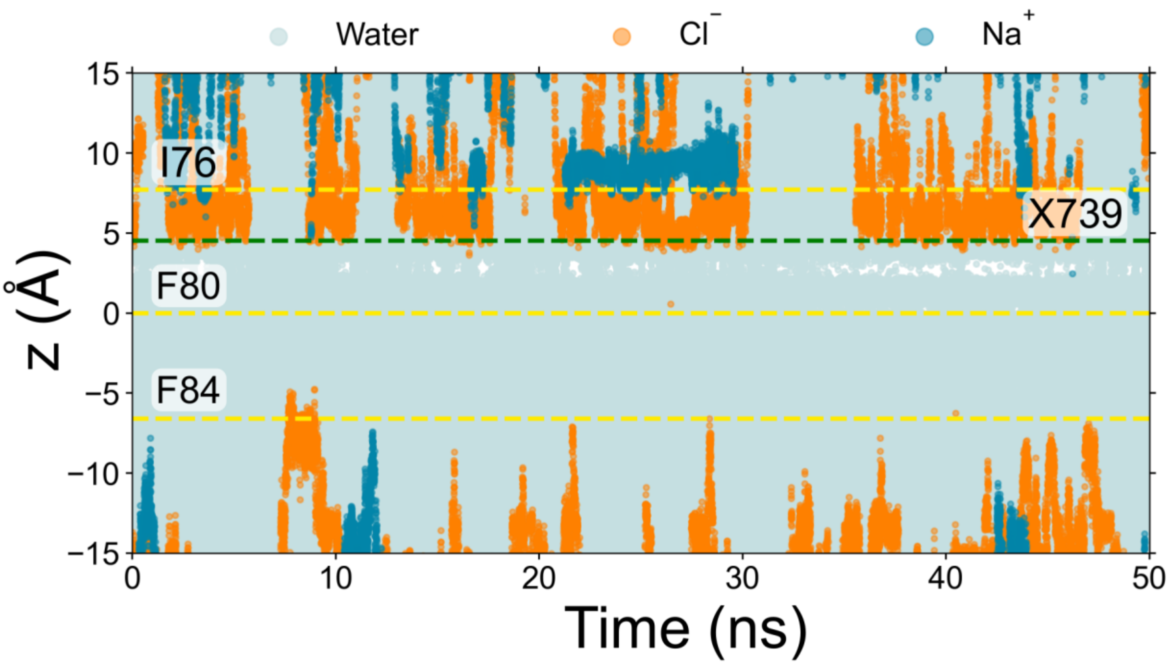

Figure S7: Replica AMOEBA simulation (of Figure 4A main text) showing trajectories of water (cyan), Na^{+} (dark blue) and Cl^{-} (orange) ions in z-coordinates as a function of time within the neck region of the partially open state (PDB ID 8D1K).

Supporting information references

1. D. H. De Jong, *et al.*, Improved parameters for the martini coarse-grained protein force field. *J Chem Theory Comput* **9**, 687–697 (2013).
2. M. J. Abraham, *et al.*, Gromacs: High performance molecular simulations through multi-level parallelism from laptops to supercomputers. *SoftwareX* **1–2**, 19–25 (2015).
3. O. N. Vickery, P. J. Stansfeld, CG2AT2: An Enhanced Fragment-Based Approach for Serial Multi-scale Molecular Dynamics Simulations. *J Chem Theory Comput* **17**, 6472–6482 (2021).
4. J. Huang, *et al.*, CHARMM36m: An improved force field for folded and intrinsically disordered proteins. *Nat Methods* **14**, 71–73 (2016).
5. D. J. Evans, B. L. Holian, The Nose-Hoover thermostat. *J Chem Phys* **83**, 4069–4074 (1985).
6. M. Parrinello, A. Rahman, Polymorphic transitions in single crystals: A new molecular dynamics method. *J Appl Phys* **52**, 7182–7190 (1981).
7. U. Essmann, *et al.*, A smooth particle mesh Ewald method. *J Chem Phys* **103**, 8577–8593 (1995).
